# Pharmacovigilance analysis of polatuzumab plus bendamustine and rituximab treatment protocol: identifying comprehensive safety signals using FDA database

**DOI:** 10.3389/fphar.2025.1459067

**Published:** 2025-02-18

**Authors:** Fang Wu, Siliang Wang, Xihui Xu, Weihui Zhang, Jie Zhou, Runyan Niu, Wenting Cai, Yonggong Yang, Mengying Liu, Jinping Zhang

**Affiliations:** ^1^ Department of Pharmacy, Nanjing Drum Tower Hospital, School of Basic Medicine and Clinical Pharmacy, China Pharmaceutical University, Nanjing, China; ^2^ Department of Pharmacy, Nanjing Drum Tower Hospital, Affiliated Hospital of Medical School, Nanjing University, Nanjing, China; ^3^ Department of Hematology, Nanjing Drum Tower Hospital, Affiliated Hospital of Medical School, Nanjing University, Nanjing, China

**Keywords:** pharmacovigilance, polatuzumab vediton, drug safety, disproportionality analysis, FAERS

## Abstract

**Background:**

The combination of polatuzumab, bendamustine and rituximab (pola+BR) was authorized for the treatment of relapsed or refractory Diffuse large B cell lymphoma (DLBCL). This study used the FDA database to identify safety signals related to the treatment protocol.

**Methods:**

The adverse events (AEs) from 2019Q1 to 2023Q3 were analyzed by calculating the reporting odds ratio. Severe and non-severe cases were compared using either an independent samples t-test or chi-squared (χ^2^) test. Additionally, a score sheet was employed to prioritize the signals.

**Results:**

In all database, 58 significant signals were detected within 1,597 patients accepting the treatment protocol. Common AEs like neutropenia, thrombocytopenia, and peripheral neuropathy, as well as other AEs like anaemia, sepsis, cytokine release syndrome and immune effector cell-associated neurotoxicity syndrome (ICANS) were a major focus. In addtion, 51.7%, 45.6% and 1.7% were sorted into low, moderate and high priority in term of clinical importance, respectively. Unexpected significant signals included intestinal obstruction, epilepsy, deep vein thrombosis, haemorrhage, increased blood lactate dehydrogenase and hypercalcemia.

**Conclusion:**

Our study identified significant AE signals for pola+BR through realworld disproportionality analysis data and analyzed the severity and clinical priority of these signals, which can assist clinicians in managing related AEs.

## 1 Introduction

Diffuse large B cell lymphoma (DLBCL) is the prevailing form of non-Hodgkin lymphoma (Vodicka et al., 2022). The classic initial treatment for DLBCL is R-CHOP, a combination of rituximab, cyclophosphamide, doxorubicin, vincristine, and prednisone. R-CHOP has the potential to cure the majority of patients. Nevertheless, approximately 40% of patients will experience treatment-resistant illness or recurrence (Tilly et al., 2022). Polatuzumab can provide patients with more effective treatment options for DLBCL. Polatuzumab specifically targets CD79b which is extensively expressed on cancerous B cells. The drug delivers monomethyl auristatin E to B cells to kill them (Deeks, 2019). Numerous global clinical trials are underway, which evaluate the efficacy of treatment protocol included polatuzumab for DLBCL (Palanca-Wessels et al., 2015; Diefenbach et al., 2021; Tilly et al., 2019).

Pola+BR was approved in 2019 for relapsed or refractory DLBCL, which was based on the pivotal trial GO29365 (Sehn et al., 2022). Pola+BR is one of the second-line treatment protocol for DLBCL in the 2023 National Comprehensive Cancer Network (NCCN) guidelines recommendations (Zelenetz et al., 2023). A few of clinical trials has evaluated the effectiveness and safety of Pola+BR, which demonstrated clinical benefit (Argnani et al., 2022; Dal et al., 2023; Wang et al., 2022). However, real-world study of the protocol has not been conducted globally by now. The FDA has developed the Food and Drug Administration Adverse Event Reporting System (FAERS) to enable people to submit reports on adverse events (AEs). Our objective was to characterize AEs of pola+BR by using the FAERS to perform large-scale post-marketing surveillance.

## 2 Patients and methods

### 2.1 The design of the study and data source

We analyzed the safety of pola+BR in B lymphoma through a comprehensive retrospective appraisement and downloaded data from DEMO, DRUG, REAC, OUTC, and INDI tables contained in the FAERS database covering the period from 2019Q1 to 2023Q3.

### 2.2 Data fetch and analysis

**FIGURE 1:**
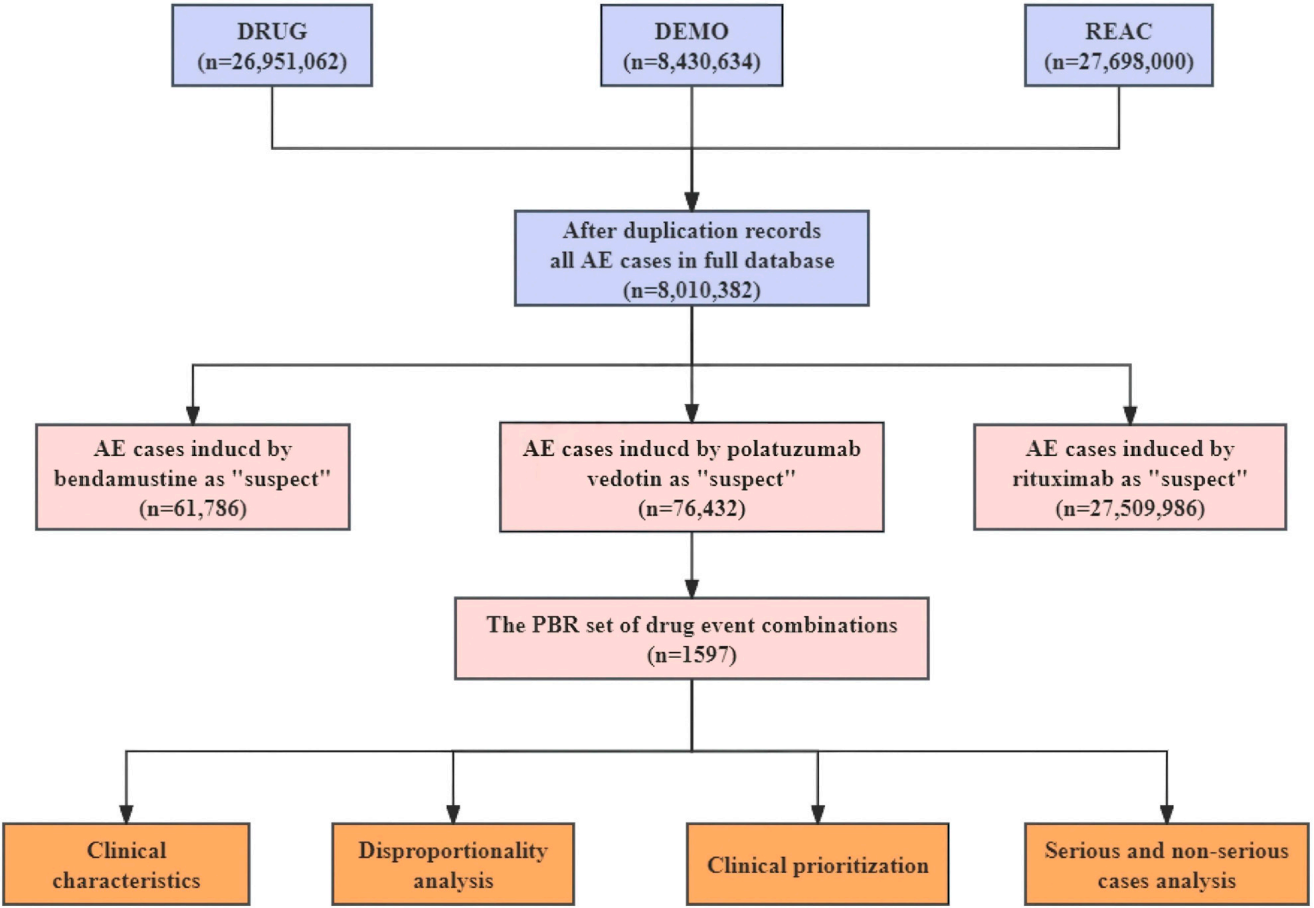
The process of extracting, processing, and analyzing data from food and drug administration adverse event reporting (FAERS) database. AE, Adverse events; Pola+BR, Polotuzumab combination Bendamustine and Rituximab.

We cleaned and merged the datasets before statistical analysis because of the duplicate reports. Initially, reports with the most recent FDA acceptance date were chosen, and repeat records were subsequently eliminated. Secondly, the study only focused on role_cod of drugs that were reported as ‘primary suspects’ or ‘secondary suspects’. Ultimately, a total of 8,010,382 cases of AEs had been uploaded to the FAERS during the study period and contained pola+BR-related AEs in 1,597 patients. The preferred terms (PTs) were categorized into System Organ Classes (SOC) based on the Medical Dictionary for regulatory Activities (MedDRA) Version 24.0. The study, serious AEs were defined as outcomes leading to hospitalizations, life-threatening illnesses, disabilities, or death (Matsumoto et al., 2023). Reporting odd ratio (ROR) represents a widely used and reliable measure of disproportionality analysis for pharmacovigilance studies based on a two-by-two contingency table, which can identify potential correlations between reported drugs and AEs. Figure 1 illustrates the procedure of extracting, processing, and analyzing data.

### 2.3 Statistical analysis

The ROR algorithm was used to detect AEs signals (Supplementary Table S1). To reduce false positives, we only retained PTs with at least 10 reports (Shu et al., 2022). A signal would be deemed significant if the lower limit of the 95% confidence interval for the ROR was greater than 1. We compared AE types between severe and non-severe cases. Comparisons were made by either a Pearson’s chi-squared (χ2) or Fisher’s exact test used for comparing proportion, while an independent samples t-test was utilized for continuous variables. By conducting a sensitivity analysis of the trend of ROR values over time to verify the robustness of the top ten signals (Zhao et al., 2023). Reports were imported and extracted by MySQL 15.0 and Navicat Premium 15, and statistical analyses were conducted with Microsoft Excel 2021 and GraphPad prism 9.

### 2.4 Clinical prioritization of signals

The prioritization of significant signals utilized a semi-quantitative score which contained factors such as the quantity of reports, ROR_025_ values, the percentage of death, classification as designated medical events (DMEs) or important medical events (IMEs), and the appraisement of evidence (Gatti et al., 2021; Guo et al., 2022). Identifying AEs with low, moderate, or high clinical priority can be done by categorizing scores as 0–4, 5–7, or 8–10. Supplementary Table S2 provides detailed information on these categories.

## 3 Results

### 3.1 Descriptive analysis

**FIGURE 2:**
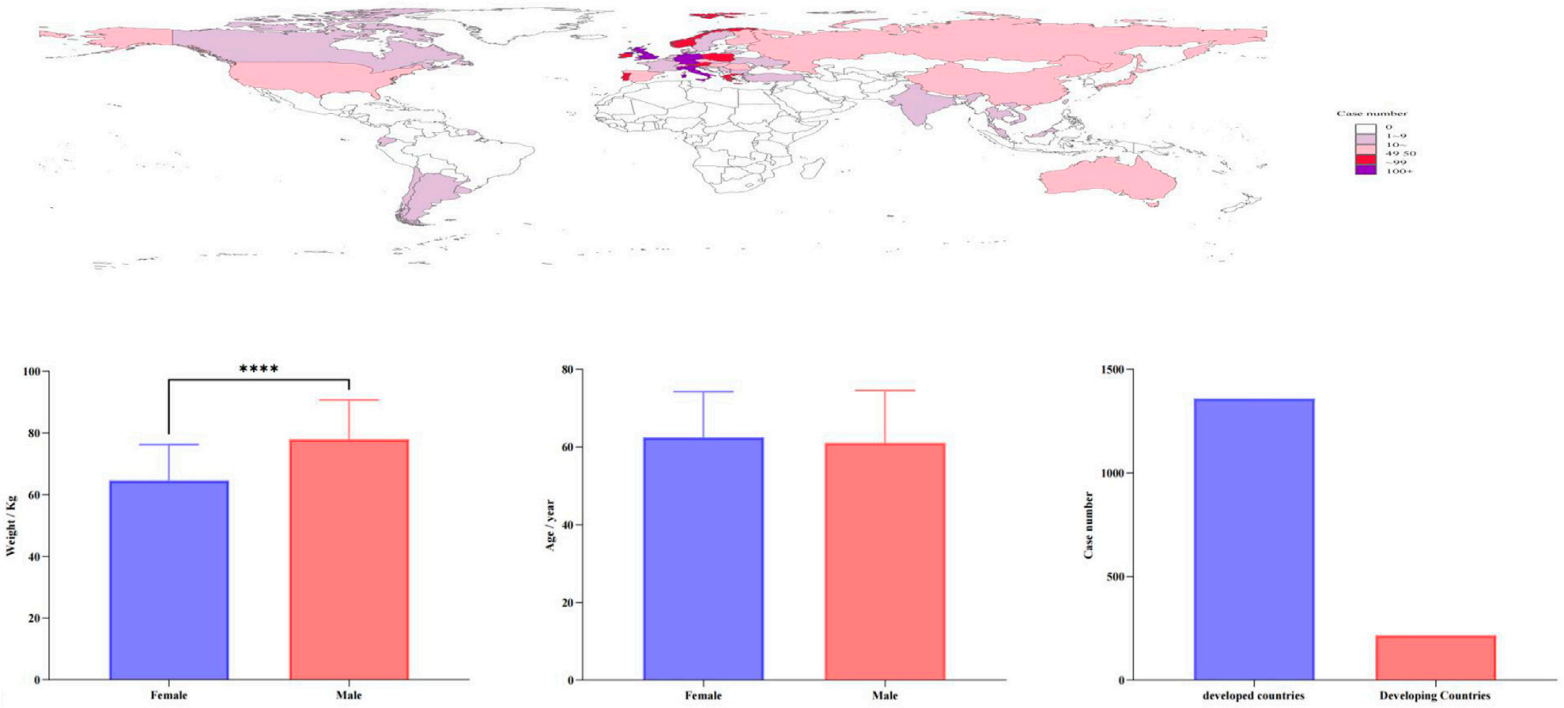
Case distribution and group characteristics of adverse event reports associated with the Pola+BR treatment protocol.

Following the completion of data cleaning, a total of 1,597 case reports with the pola+BR treatment protocol were collected between January 2019 and September 2023. The comprehensive clinical characteristics can be displayed in Table 1 and Figure 2. The proportion of patients using pola+BR was higher among males (43.71%) than females (31.06%), and 29.68% patients exprienced serious outcomes. Notablely, 83% of the patients were diagnosed with DLBCL and the number of patients using this treatment regimen has increased year by year.

**TABLE 1:** Characteristics of adverse events reports associated with the pola+BR treatment protocol. From 2019Q1 to 2023Q3.

Characteristics	Pola+BR (N = 1,597)
Gender, n (%)	
Female	496 (31.06%)
Male	698 (43.71%)
Unknown	385 (24.11%)
Age (years), n (%)	
<18	2 (0.13%)
18≤and≤65	414 (25.92%)
>65	354 (22.17%)
Unknown	809 (50.66%)
Reported countries, n (%)	
United States	33 (2.07%)
Italy	213 (13.34%)
Canada	6 (0.38%)
Great Britain	323 (20.23%)
Germany	262 (16.41%%)
Others	760 (47.59%)
Indications, n (%)	
DLBCL	1,249 (78.21%)
DLBCL refractory	59 (3.69%)
DLBCL recurrent	24 (1.50%)
Others	265 (16.59%)
Outcomes, n (%)	
Non-serious outcome	1,123 (70.32%)
Serious outcome	474 (29.68%)
Report year, n (%)	
2019	1 (0.06%)
2020	23 (1.44%)
2021	108 (6.76)
2022	476 (29.81%)
2023	971 (60.80%)

### 3.2 Disproportionality analysis

**FIGURE 3:**
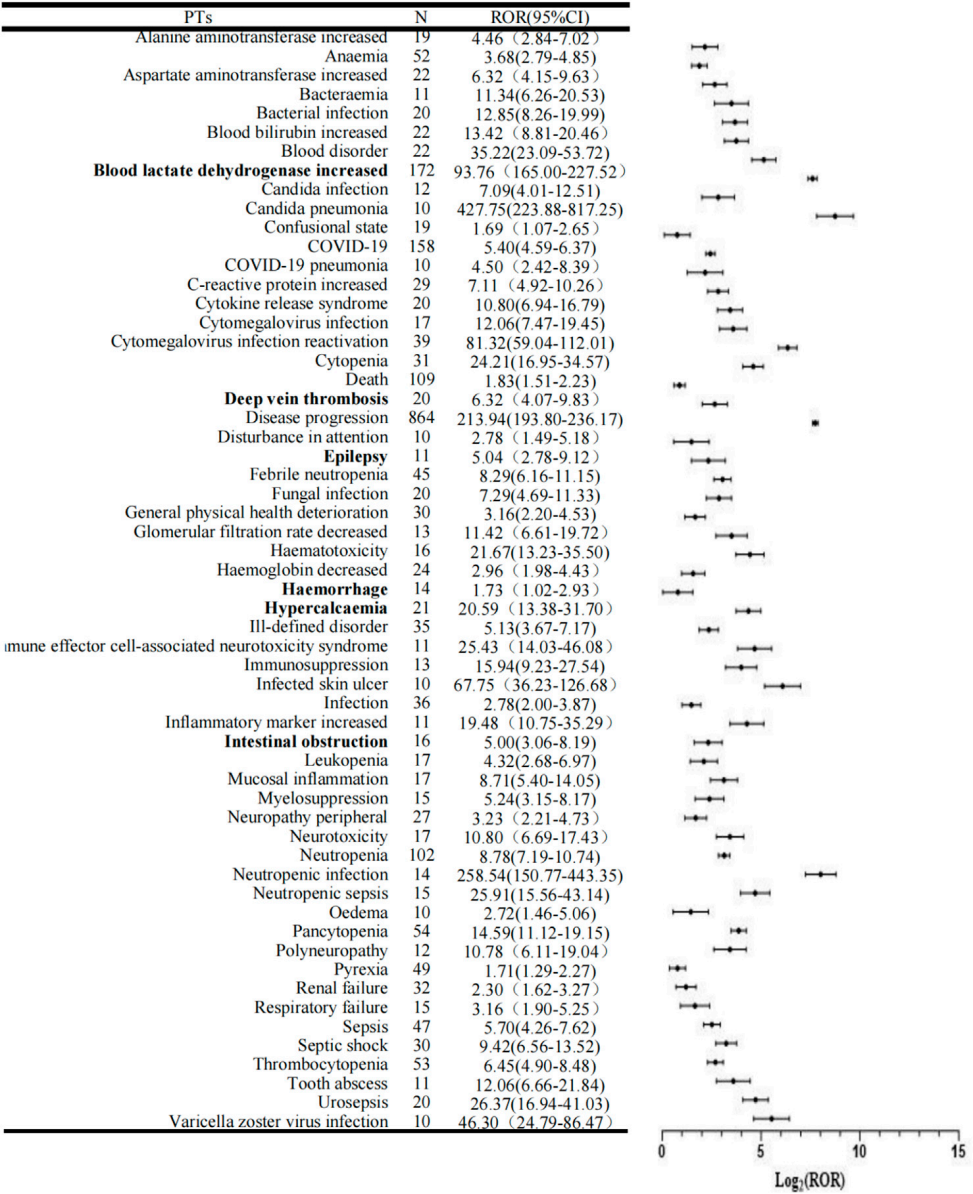
Forest plots of disproportionality of the Pola+BR treatment protocol. PTs, preferred terms; N, number of cases; ROR, Reporting Odds Ration; CI, Confidence Interval.

There were 58 significant PTs in 11 SOCs shown in Table 2 and Figure 3. In addition PTs with fewer than 10 reports were listed in Supplementary Table S3. COVID-19, neutropenia, pancytopenia, thrombocytopenia and anaemia were the most common AEs besides disease progress, death and blood lactate dehydrogenate. Unexpected adverse events (AEs) that were not identified in previous clinical studies and instructions were classified for 6 PTs, such as intestinal obstruction, epilepsy, deep vein thrombosis, haemorrhage, increased blood lactate dehydrogenase and hypercalcemia.

**TABLE 2:** Disproportionate distribution of positive signals of the pola+BR treatment protocol.

SOC	PT	N	ROR (95%CI)
Blood and lymphatic system disorders	Neutropenia	102	8.78 (7.19–10.74)
	Pancytopenia	54	14.59 (11.12–19.15)
	Thrombocytopenia	53	6.45 (4.90–8.48)
	Anaemia	52	3.68 (2.79–4.85)
	Febrile neutropenia	45	8.29 (6.16–11.15)
	Cytopenia	31	24.21 (16.95–34.57)
	Blood disorder	22	35.22 (23.09–53.72)
	Leukopenia	17	4.32 (2.68–6.97)
	Haematotoxicity	16	21.67 (13.23–35.50)
	Myelosuppression	15	5.24 (3.15–8.17)
Gastrointestinal disorders	**Intestinal obstruction**	16	5.00 (3.06–8.19)
General disorders and administration site conditions	Disease progression	864	213.94 (193.80–236.17)
	Death	109	1.83 (1.51–2.23)
	Pyrexia	49	1.71 (1.29–2.27)
	Ill-defined disorder	35	5.13 (3.67–7.17)
	General physical health deterioration	30	3.16 (2.20–4.53)
	Mucosal inflammation	17	8.71 (5.40–14.05)
	Oedema	10	2.72 (1.46–5.06)
Immune system disorders	Cytokine release syndrome	20	10.80 (6.94–16.79)
	Immunosuppression	13	15.94 (9.23–27.54)
Infections and infestations	COVID-19	158	5.40 (4.59–6.37)
	Sepsis	47	5.70 (4.26–7.62)
	Cytomegalovirus infection reactivation	39	81.32 (59.04–112.01)
	Infection	36	2.78 (2.00–3.87)
	Septic shock	30	9.42 (6.56–13.52)
	Bacterial infection	20	12.85 (8.26–19.99)
	Fungal infection	20	7.29 (4.69–11.33)
	Urosepsis	20	26.37 (16.94–41.03)
	Cytomegalovirus infection	17	12.06 (7.47–19.45)
	Neutropenic sepsis	15	25.91 (15.56–43.14)
	Neutropenic infection	14	258.54 (150.77–443.35)
	*Candida* infection	12	7.09 (4.01–12.51)
	Bacteraemia	11	11.34 (6.26–20.53)
	Tooth abscess	11	12.06 (6.66–21.84)
	*Candida* pneumonia	10	427.75 (223.88–817.25)
	COVID-19 pneumonia	10	4.50 (2.42–8.39)
	Infected skin ulcer	10	67.75 (36.23–126.68)
	Varicella zoster virus infection	10	46.30 (24.79–86.47)
Investigations	**Blood lactate dehydrogenase increased**	172	193.76 (165.00–227.52)
	C-reactive protein increased	29	7.11 (4.92–10.26)
	Haemoglobin decreased	24	2.96 (1.98–4.43)
	Aspartate aminotransferase increased	22	6.32 (4.15–9.63)
	Blood bilirubin increased	22	13.42 (8.81–20.46)
	Alanine aminotransferase increased	19	4.46 (2.84–7.02)
	Glomerular filtration rate decreased	13	11.42 (6.61–19.72)
	Inflammatory marker increased	11	19.48 (10.75–35.29)
Metabolism and nutrition disorders	**Hypercalcaemia**	21	20.59 (13.38–31.70)
Nervous system disorders	Neuropathy peripheral	27	3.23 (2.21–4.73)
	Confusional state	19	1.69 (1.07–2.65)
	Neurotoxicity	17	10.80 (6.69–17.43)
	Polyneuropathy	12	10.78 (6.11–19.04)
	**Epilepsy**	11	5.04 (2.78–9.12)
	Immune effector cell-associated neurotoxicity syndrome	11	25.43 (14.03–46.08)
	Disturbance in attention	10	2.78 (1.49–5.18)
Renal and urinary disorders	Renal failure	32	2.30 (1.62–3.27)
Respiratory.thoracic and mediastinal disorders	Respiratory failure	15	3.16 (1.90–5.25)
Vascular disorders	**Deep vein thrombosis**	20	6.32 (4.07–9.83)
	**Haemorrhage**	14	1.73 (1.02–2.93)

Unexpected signals are in bold.

SOC, system organ classes; PTs, preferred terms; N, number of cases; ROR, reporting odds ration; CI, confidence interval.

### 3.3 Clinical prioritization of significant signals

Clinical prioritization of AEs was summarized in Table 3. All together, 26 out of 58 PTs (40.63%) were categorized as IMEs. A total of 5 PTs (7.81%) were identified as DMEs, including pancytopenia, febrile neutropenia, neutropenic infection, neutropenic sepsis, and renal failure. On the basis of clinical priority score, PTs were sorted into low, moderate, and high clinical priority, comprising 30 (51.7%), 27 (46.6%), and one (1.7%) respectively. Pancytopenia (score 8) emerged as high clinical priorities. Neutropenia, thrombocytopenia, anaemia, febrile neutropenia, intestinal obstruction, cytokine release syndrome (CRS), sepsis, cytomegalovirus infection reactivation, neurotoxicity and polyneuropathy were graded as moderate clinical prioporities. Among the 27 adverse events identified as moderate clinical priorities were conditions like neutropenia, thrombocytopenia, anaemia, febrile neutropenia, intestinal obstruction, CRS, sepsis, cytomegalovirus infection reactivation, neurotoxicity, polyneuropathy, and so on. With the evidence evaluated, it was determined that 22 AEs showed high clinical evidence with a rating of “++.”

**TABLE 3:** Clinical priority assessing results of positive signals.

SOC	PT	n	ROR025	Death (n)	IME/DME	Relevant evidence evation	Priority level (score)
Blood and lymphatic system disorders	Neutropenia	102	7.19	20	IME	++	Moderate (7)
	Pancytopenia	54	11.12	17	DEM	++	High (8)
	Thrombocytopenia	53	4.90	10	IME	++	Moderate (6)
	Anaemia	52	2.79	14	NA	++	Moderate (5)
	Febrile neutropenia	45	6.16	12	DEM	++	Moderate (7)
	Cytopenia	31	16.95	2	IME	+	Moderate (5)
	Blood disorder	22	23.09	3	NA	++	Moderate (5)
	Leukopenia	17	2.68	3	IME	+	Low (4)
	Haematotoxicity	16	13.23	0	NA	++	Moderate (5)
	Myelosuppression	15	3.15	0	IME	++	Moderate (5)
Gastrointestinal disorders	Intestinal obstruction	16	3.06	0	IME	+	Low (4)
General disorders and administration site conditions	Disease progression	864	193.80	120	NA	—	Moderate (5)
	Death	109	1.51	109	IME	—	Low (4)
	Pyrexia	49	1.29	8	NA	++	Low (3)
	Ill-defined disorder	35	3.67	7	NA	−	Low (2)
	General physical health deterioration	30	2.20	8	NA	+	Low (3)
	Mucosal inflammation	17	5.40	0	NA	+	Low (4)
	Oedema	10	1.46	3	NA	++	Low (3)
Immune system disorders	Cytokine release syndrome	20	6.94	0	IME	+	Moderate (5)
	Immunosuppression	13	9.23	1	IME	+	Moderate (5)
Infections and infestations	COVID-19	158	4.59	47	NA	+	Low (4)
	Sepsis	47	4.26	16	IME	++	Moderate (5)
	Cytomegalovirus infection reactivation	39	59.04	2	IME	+	Moderate (5)
	Infection	36	2.00	12	NA	++	Low (4)
	Septic shock	30	6.56	15	IME	++	Moderate (6)
	Urosepsis	20	16.94	11	IME	+	Moderate (5)
	Bacterial infection	20	8.26	14	NA	+	Low (4)
	Fungal infection	20	4.69	2	NA	+	Low (3)
	Cytomegalovirus infection	17	7.47	2	IME	+	Moderate (5)
	Neutropenic sepsis	15	15.56	0	DEM	++	Moderate (7)
	Neutropenic infection	14	150.77	0	DEM	++	Moderate (7)
	*Candida* infection	12	4.01	8	NA	+	Low (3)
	Bacteraemia	11	6.26	11	IME	+	Moderate (5)
	Tooth abscess	11	6.66	0	IME	+	Moderate (5)
	COVID-19 pneumonia	10	2.42	7	IME	++	Moderate (5)
	*Candida* pneumonia	10	223.88	0	IME	+	Moderate (5)
	Infected skin ulcer	10	36.23	0	NA	+	Low (4)
	Varicella zoster virus infection	10	24.79	1	NA	+	Low (4)
Investigations	Blood lactate dehydrogenase increased	172	165.00	36	NA	+	Moderate (5)
	C-reactive protein increased	29	4.92	8	NA	+	Low (3)
	Haemoglobin decreased	24	1.98	0	NA	++	Low (3)
	Aspartate aminotransferase increased	22	4.15	5	NA	++	Low (4)
	Blood bilirubin increased	22	8.81	0	NA	—	Low (3)
	Alanine aminotransferase increased	19	2.84	5	NA	++	Low (4)
	Glomerular filtration rate decreased	13	6.61	1	NA	+	Low (4)
	Inflammatory marker increased	11	10.75	0	NA	+	Low (4)
Metabolism and nutrition disorders	Hypercalcaemia	21	13.38	5	NA	+	Low (4)
Nervous system disorders	Neuropathy peripheral	27	2.21	7	IME	++	Moderate (5)
	Confusional state	19	1.07	8	NA	+	Low (2)
	Neurotoxicity	17	6.69	0	IME	++	Moderate (6)
	Polyneuropathy	12	6.11	0	IME	+	Moderate (5)
	Immune effector cell-associated neurotoxicity syndrome	11	14.03	1	IME	+	Moderate (5)
	Epilepsy	11	2.78	0	IME	+	Low (4)
	Disturbance in attention	10	1.49	5	NA	+	Low (2)
Renal and urinary disorders	Renal failure	32	1.62	12	DEM	+	Low (4)
respiratory.thoracic and mediastinal disorders	Respiratory failure	15	1.90	2	IME	+	Low (3)
Vascular disorders	Deep vein thrombosis	20	4.07	1	IME	+	Low (4)
	Haemorrhage	14	1.02	5	IME	++	low (4)

A priority score between 8 and 10, 5–7 or 0–4 represents the signal with high, moderate or low clinical priority, respectively. NA, Not Applicable (for relevant criterias); n, number of cases; SOC, System Organ Classes; PTs, Preferred Terms; ROR_025_, the lower limit of 95% confidence interval of ROR; IME, important medical events; DME, designated medical events.

### 3.4 Contrasting severe and non-severe cases

The study included 1,597 patients of whom 474 had serious outcomes. Table 4 displays a statistically significant difference in gender (*p* = 0.02) between severe and non-severe cases. Males (32.23%) had a higher rate of serious AEs compared to females (26.01%). By contrast, age (*p* = 0.922) and weight (*p* = 0.608) did not differ between the two groups. With a p-value of less than 0.05, 42 PTs were more prone to be identified as serious AEs, including anaemia, febrile neutropenia, thrombocytopenia, intestinal obstruction, pyrexia, CRS, COVID-19, sepsis, neuropathy peripheral, epilepsy, renal failure and deep vein thrombosis. Additionally, other PTs showed a tendency to be classified into non-severe AEs with a p-value greater than 0.05, such as pancytopenia, oedema, aspartate aminotransferase increased and alanine aminotransferase increased.

**TABLE 4:** Differences in clinical characteristics of severe and non-severe reports.

	Serious cases	Non-serious cases	Statistic	p-value
Age, years (Mean ± SD)	61.05 ± 14.02	58 ± 13.04	0.10^c^	0.922
weight, kg (Mean ± SD)	73.72 ± 16.4	73.62 ± 13.32	0.51^c^	0.608
Sex distribution, n				
**Male**	**225**	**473**	**5.39** ^d^	**0.020** ^a^
Female	129	367		
Types of AEs, n				
**Anaemia**	**26**	**26**	**10.633** ^d^	**0.001** ^a^
**Febrile neutropenia**	**26**	**19**	**33.710** ^d^	**<0.001** ^a^
**Thrombocytopenia**	**26**	**27**	**9.861** ^d^	**0.002** ^a^
**Haematotoxicity**	**15**	**1**	**31.787** ^d^	**<0.001** ^a^
**Leukopenia**	**12**	**5**	**13.777** ^d^	<**0.001** ^a^
Neutropenia	37	65	2.270^d^	0.131^a^
Pancytopenia	31	23	3.706^d^	0.054^a^
Cytopenia	14	17	3.630^d^	0.057^a^
Myelosuppression	6	9	0.773^d^	0.379^a^
Blood disorder	3	19	2.751^d^	0.097^a^
**Intestinal obstruction**	**0**	**16**	**—**	**0.005** ^b^
**Disease progression**	**202**	**662**	**35.809** ^d^	**<0.001** ^a^
**Death**	**109**	**0**	**277.160** ^d^	**<0.001** ^a^
**Pyrexia**	**40**	**9**	**65.371** ^d^	**<0.001** ^a^
**General physical health deterioration**	**17**	**13**	**10.668** ^d^	**0.001** ^a^
**Mucosal inflammation**	**14**	**3**	**22.841** ^d^	**<0.001** ^a^
Ill-defined disorder	7	28	1.607^d^	0.205^a^
Oedema	3	7	—	0.982^b^
**Cytokine release syndrome**	**17**	**3**	**29.697** ^d^	**<0.001** ^a^
**Immunosuppression**	**13**	**0**	**—**	**<0.001** ^b^
**COVID-19**	**79**	**79**	**34.687** ^d^	**<0.001** ^a^
**Sepsis**	**32**	**15**	**34.220** ^d^	**<0.001** ^a^
**Septic shock**	**28**	**2**	**59.353** ^d^	**<0.001** ^a^
**Infection**	**17**	**19**	**5.430** ^d^	**0.020** ^a^
**Bacterial infection**	**14**	**6**	**15.775** ^d^	**<0.001** ^a^
**Fungal infection**	**12**	**8**	**8.921** ^d^	**0.003** ^a^
**Urosepsis**	**11**	**9**	**6.221** ^d^	**0.013** ^a^
**Candida infection**	**11**	**1**	**—**	**<0.001** ^b^
**Bacteraemia**	**11**	**0**	**—**	**<0.001** ^b^
**Tooth abscess**	**11**	**0**	**—**	**<0.001** ^b^
**Infected skin ulcer**	**10**	**0**	**—**	**<0.001** ^b^
**Cytomegalovirus infection**	**9**	**8**	**4.454** ^d^	**0.035** ^a^
**COVID-19 pneumonia**	**7**	**3**	**—**	**0.010** ^b^
**Cytomegalovirus infection reactivation**	**2**	**37**	**11.546** ^d^	**<0.001** ^a^
**Neutropenic sepsis**	**0**	**15**	**—**	**0.008** ^b^
**Neutropenic infection**	**0**	**14**	**—**	**0.014** ^b^
*Candida* pneumonia	4	6	—	0.495^b^
Varicella zoster virus infection	2	8	—	0.732^b^
**C-reactive protein increased**	**15**	**14**	**6.877** ^d^	**0.009** ^a^
**Inflammatory marker increased**	**11**	**0**	**—**	**<0.001** ^b^
**Haemoglobin decreased**	**2**	**22**	**5.320** ^d^	**0.021** ^a^
**Blood bilirubin increased**	**0**	**22**	**9.416** ^d^	**0.002** ^a^
Blood lactate dehydrogenase increased	48	124	0.291^d^	0.590^a^
Aspartate aminotransferase increased	5	17	0.517^d^	0.472^a^
Alanine aminotransferase increased	5	14	0.104^d^	0.747^a^
Glomerular filtration rate decreased	1	12	—	0.124^b^
**Hypercalcaemia**	**11**	**10**	**5.254** ^d^	**0.022** ^a^
**Neuropathy peripheral**	**14**	**13**	**6.468** ^d^	**0.011** ^a^
**Confusional state**	**13**	**6**	**13.827** ^d^	**0.002** ^a^
**Epilepsy**	**11**	**0**	**—**	**<0.001** ^b^
**Immune effector cell-associated neurotoxicity syndrome**	**11**	**0**	**—**	**<0.001** ^b^
**Polyneuropathy**	**0**	**12**	**—**	**0.023** ^b^
Disturbance in attention	5	5	—	0.173^b^
Neurotoxicity	2	15	2.643^d^	0.104^a^
**Renal failure**	**20**	**12**	**16.852** ^d^	**<0.001** ^a^
Respiratory failure	6	9	—	0.399^b^
**Deep vein thrombosis**	**1**	**19**	**5.911** ^d^	**0.015** ^a^
**Haemorrhage**	**9**	**5**	**—**	**0.007** ^b^

Results that are statistically significant are in bold.

^a^ Proportions were compared using Pearson χ2 test.

^b^ Fisher’s exact test.

^c^ The t-statistic of the independent samples t-test.

^d^ The χ2 value of the Pearson chi-square test.

AEs, Adverse Events; n, number of cases.

### 3.5 Sensitivity analysis

**FIGURE 4:**
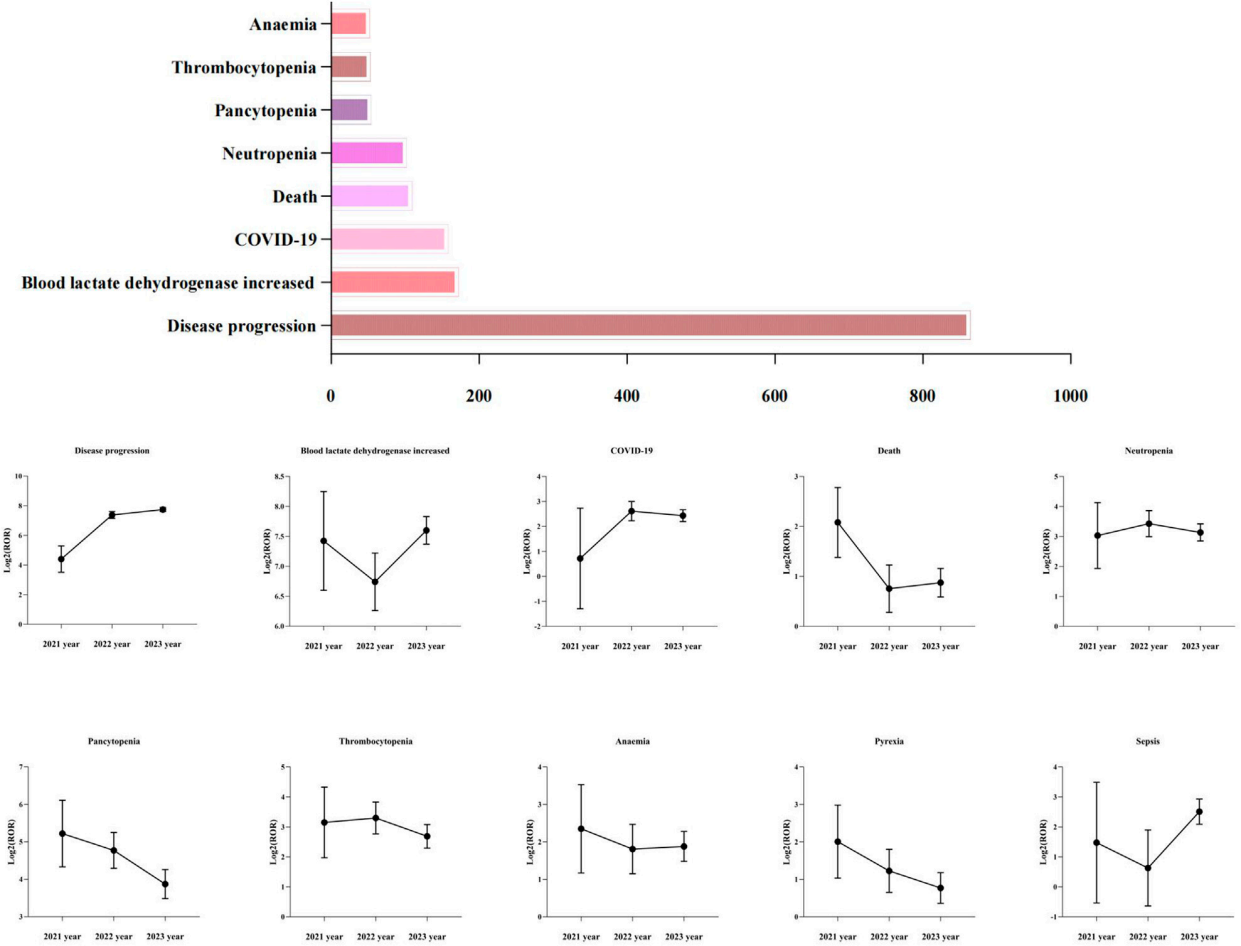
The top ten positive signals of case number and their sensitivity analysis. ROR, Reporting Odds Ration.

To reduce the risk of false positives in AEs detection and confirm the stability of the signals, this study conducted a sensitivity analysis on the top ten positive signals by case report number. By calculating the reporting ROR and its 95% confidence interval corresponding to the annual cumulative case volume of disease progression, blood lactate dehydrogenase increased, COVID-19, death, neutropenia, pancytopenia, thrombocytopenia, anaemia, pyrexia and sepsis, we assessed the trend of the signals over time, as detailed in Figure 4. As the number of cases continues to accumulate, the 95% confidence intervals for the ROR values of these 10 positive signals gradually narrow and stabilize, further validating the robustness of the signals.

## 4 Discussion

The most recent safety profiles of the pola+BR treatment protocol were examined in this study through post-marketing analysis by using data from the FAERS. We found that the number of patients taking pola+BR has increased year by year and more patients will possibly choose the pola+BR regimen in the future. Therefore, it is important to comprehensively monitor AEs in this regimen. As shown in Figure 2, the reports of related AEs of Pola + BR were mainly concentrated in developed countries, and the reasons may involve two main aspects: First, the FAERS database is a system relying on spontaneous reporting. In addition to FAERS, there are other databases such as Vigibase and Japanese Adverse Drug Event Reporting System, which may lead to the FAERS collected reports mainly from European and American countries. Second, UGT1A1 gene polymorphisms closely related to the drug metabolism of antibody-conjugated drugs were associated with the occurrence of treatment-related AEs, while UGT1A1 expression varied across ethnic groups (Tarantino et al., 2023). Together, these factors may contribute to the geographical imbalance in the reporting of Pola + BR-related AEs. According to our research, males (32.23%) were prone to exhibit serious AEs. In accordance with epidemiologic studies of DLBCL, pola+BR-associated AEs were more common in males (43.71%) than females (31.06%). This phenomenon may also be associated with body weight, as illustrated in Figure 2, which demonstrates a significant difference in body weight between male and female patient groups experiencing AEs related to pola+BR. Additionally, studies (Gibiansky et al., 2022; Yin et al., 2021) have shown that changes in body weight affect the pharmacokinetics of certain antibody-drug conjugates. Our findings indicated that there was no disparity in body weight between severe and non-severe instances. However, a time-to-event analysis study (Lu et al., 2017) on polatizumab discovered that body weight served as a prognostic indicator for secondary peripheral neuropathy in patients treated with polatizumab.

Polatuzumab received approval in June 2019, which was due to the outcomes of the clinical trial GO29365 (3). The most reported AEs in patients accepting pola+BR were neutropenia, diarrhea, nausea, thrombocytopenia and peripheral neuropathy (>30%). In the clinical trial GO29365, 41.7% of pola+BR patients reported serious AEs, and the most serious AEs were febrile neutropenia, sepsis, infectious pneumonia and pyrexia occurring in more than 5% of cases. In our study, neutropenia and thrombocytopenia were involved in the most reported AEs. In the pola+BR group, 29.68% of patients experienced serious adverse events (AEs), including febrile neutropenia, sepsis, and COVID-19 pneumonia. This consistency further substantiates the reliability of our research findings. Myelosuppression, periphral neuropathy, and infusion-related reactions led to dose reductions or discontinuation according to the polatuzumab vedotin labeling. In the pivotal clinical trial GO29365, treatment was terminated on account of thrombocytopenia (>5%), neutropenia (>4%), periphral neuropathy (2.6%) and infection (2.6%) among patients treated with pola+BR. In our study, thrombocytopenia, anaemia, febrile neutropenia, cytomegalovirus infection reactivation and immunosuppression were rated as moderate clinical priority and all of these were reported as serious AEs more possibly. It manifests that patients accepting pola+BR need adequate supportive care, such as transfusion support, growth factors infusion, appropriate antimicrobial prophylaxis and monitoring for infections (Smith et al., 2021).

At present, sepsis and the septic shock are confronting important clinical problems in the field of acute critical care medicine. A new extension cohort study (Sehn et al., 2022) of pola+BR in relapsed/refractory DLBCL, 9.9% patients discontinued treatment due to serious sepsis in the pooled pola+BR cohort. In our study, sepsis, sepsis shock and neutropenic sepsis were rated as moderate clinical priority and prone to be listed in serious AEs. An analysis (Xia et al., 2022) of drug safety using the FAERS database revealed 35 cases of sepsis, 21 cases of sepsis shock and 8 cases of neutropenic sepsis associated with polatuzumab vedotin from the first quarter of 2004 to the third quarter of 2021. The ROR of polatuzumab inducing sepsis-related AEs is 8.30, which suggests that polatuzumab increases the risk of sepsis-related AEs. Clinicians should be alert to sepsis-related AEs when pola+BR is applied to patients. Initiatives such as early recognition, severity assessment and early therapy (antimicrobials and hemodynamic optimization) are beneficial to reduce both morbidity and mortality of sepsis (Jouffroy et al., 2024). In clinical practice, dynamic monitoring of early warning scoring systems such as the National Early Warning Score (NEWS), Sequential Organ Failure Assessment (SOFA) score, and Multisystem Organ Dysfunction Syndrome (MODS) severity score are crucial for the early identification and intervention of sepsis. Studies have shown that the NEWS score performs well in identifying high-risk patients, particularly in non-critical care units, with both high sensitivity and specificity (Pullyblank et al., 2020). Additionally, the SOFA score is widely used to assess the organ function status of sepsis patients, effectively predicting mortality rates during hospitalization (Lambden et al., 2019). This multidimensional evaluation method provides clinicians with more precise decision-making support, contributing to better patient outcomes in sepsis management.

Over the past decades, numerous innovative medications have received approval in the fields of oncology and hematology. Cancer immunotherapy has progressed rapidly in recent years. Efficient immunotherapies such as anti-CD20 monoclonal antibody (rituximab) and antibodies against CD79b (polatuzumab) have already been approved for DLBCL (Roth et al., 2021). However, these powerful immunotherapeutic drugs are also linked to potentially deadly side effects—particularly disorders of the immune system, which are drawing attention alongside clinical application experience. CRS and ICANS (Freyer and Porter, 2020) are the most frequent immune-related toxicites. CRS presents typically as pyrexia, fatigue, loss of appetite and so on, but in severe cases, it can also lead to low blood pressure, oxygen deficiency, and/or organ dysfunction (Shimabukuro-Vornhagen et al., 2018). ICANS usually manifests toxic brain disorder, difficulties in speech, confusion, and in more severe cases, seizures, amyosthenia, and brain swelling have been observed (Gu et al., 2022). Patients with ICANS almost always have a history of CRS before developing ICANS, and ICANS usually occurs after CRS remission (Morris et al., 2022). Eleven patients who presented ICANS in our study had also experienced CRS. While CRS and ICANS are serious AEs, most symptoms that do not cause permanent harm can be resolved. Therefore, it is of great importance to identify and manage CRS and ICANS. In the early identification of neurotoxicity, it is crucial to promptly monitor for neurological symptoms in patients during the treatment process. These symptoms may include pain, numbness, dizziness, and so on. Medical professionals should remain vigilant about the potential risk of neurotoxicity associated with Pola+BR and stop treatment in a timely manner upon detection of relevant symptoms to prevent further neurological damage.

Unexpected safety signals included epilepsy, intestinal obstruction, deep vein thrombosis, haemorrhage, blood lactate dehydrogenase increased and hypercalcaemia. In three case reports (Haefner et al., 2007; Hosoi et al., 2010; Jennane et al., 2022), the patients treated with a rituximab-containing regimen presented generalized seizures. All of them developed posterior reversible encephalopathy syndrome following MRI examination. This suggests that when epilepsy occurs in patients with pola+BR, a rare reversible encephalopathy syndrome caused by rituximab in this regimen may emerge. Recent studies have indicated that Polatuzumab Vedotin may elicit immune responses which can affect the stability of the nervous system and potentially lead to neurological complications such as seizures. The mechanisms underlying these immune responses are not yet fully understood, but research (Broekaart et al., 2018) suggests that activation of the immune system may be associated with seizures and disease progression, particularly in cases of where there is a disruption in the blood-brain barrier function. In addition, the use of Polatuzumab Vedotin may be associated with the onset of other autoimmune diseases. For example, some patients treated with this drug have developed symptoms similar to those of autoimmune myositis, which may further burden the nervous system (Kamo et al., 2019). These observations suggest that when using Polatuzumab Vedotin clinically, doctors should closely monitor neurological symptoms in patients, especially those with a history of epilepsy or other neurological disorders. In a clinical analysis (Kasi et al., 2012) of rituximab, the most widespread gastrointestinal symptoms were bowel obstruction and perforations, typically occurring 6 days after treatment. Studies have shown that damage to the intestinal mucosa is associated with multiple factors, including the direct effects of the drug, dysbiosis of the gut microbiota, and abnormal immune system responses. For example, certain chemotherapeutic agents have been shown to cause dysbiosis of the gut microbiota, leading to intestinal inflammation and dysfunction, which may share similarities with the mechanism of action of Polatuzumab Vedotin (Huang et al., 2022). In another clinical trial of polatuzumab (Lynch et al., 2023), two patients experienced deep vein thrombosis. In another clinical trial of polatuzumab (Wang et al., 2022), one patient experienced intracranial hemorrhage. Blood lactate dehydrogenase increased and hypercalcaemia are likely to be relative to the disease itself or the disease progression.

In this study, there were several limitations. Initially, data submitted to FAERS were incomplete, and not all adverse reports were uploaded to FAERS. Therefore, the incidence of identified risks could not be quantified accurately (Noguchi et al., 2021). Before conducting AEs retrieval, we standardized the drug names using MedDRA terminology, covering the brand names, trade names, and generic names of the drugs, among others. This step ensured that the data we collected was as comprehensive as possible, thereby mitigating the impact of missing data. Additionally, reporting biases can exist, because people prefer to reporting relatively serious AEs. Although the FAERS database compiles global data, the reports are primarily concentrated in European and American countries, with relatively fewer reports from other regions. It is noteworthy that different racial groups may have varying sensitivities to the Pola+BR regimen, which could lead to different AE manifestations. Ultimately, the study considered the pola+BR treatment regimen as a unified entity, which makes it difficult to ascertain the impact of individual drug on the identified signals. Therefore, it is necessary to conduct large-scale prospective clinical studies to address questions that the FAERS database cannot answer, thereby optimizing the rational use of clinical medication.

## 5 Conclusion

Our pharmacovigilance study analyzes real-world large-sample safety data to determine the correlation between the pola+BR treatment protocol and AEs. From 2019Q1 to 2023Q3, reports regarding the pola+BR treatment protocol increase by years. Out of the 58 identified significant signals, thrombocytopenia, anaemia, febrile neutropenia, sepsis, neuropathy peripheral, CRS and ICANS should be highly concerned. Of note, 6 PTs--epilepsy, intestinal obstruction, deep vein thrombosis, haemorrhage, blood lactate dehydrogenase increased and hypercalcaemia were new unexpect signals. In addition, 1, 27, and 30 AEs were sorted into high, moderate, and low clinical priorities. Our study enhances comprehension of the safety characteristics of pola+BR, which will assist medical practitioners in handling associated AEs during clinical practice.

## Data Availability

The raw data supporting the conclusions of this article will be made available by the authors, without undue reservation.
