# Supplementary material for: Pharmacovigilance analysis of polatuzumab plus bendamustine and rituximab treatment protocol: identifying comprehensive safety signals using FDA database
Source: Front Pharmacol. 2025 Feb 18;16:1459067. doi: 10.3389/fphar.2025.1459067 (PMC11876141; doi:10.3389/fphar.2025.1459067)
Supplement: Supplementary file 1 [file Table1.docx]

**Supplementary Table S1**. Calculation of reporting odds ratio (ROR).

|  | Reports with target AE | Reports without target AE |
| --- | --- | --- |
| Reports with pola+BR | a | b |
| Reports without pola+BR | c | d |

a, number of reports containing both the target drug and target AE; b, number of reports containing other AEs of the target drug; c, number of reports containing the target AE of other drugs; d, number of reports containing other drugs and other AEs.

AEs, Adverse Events; ROR, Reporting Odds Ratio; CI, confidence interval.

The calculation formulas are shown below:

1. ROR=ad/b/c
2. 95%CI=e^ln(ROR)±1.96(1/a+1/b+1/c+1/d)^0.5^

**Supplementary Table S2**. A rating scale assessing clinical priority of disproportionality signals.

| **Assessment items** | **2 points** | **1 point** | **0 point** |
| --- | --- | --- | --- |
| Number of target events | >50 | 10-50 | <10 |
| ROR_025_ | >5 | 2-5 | 1-2 |
| Mortality proportion | >50% | 25-50% | <25% |
| IMEs or DMEs | DME | IME | None |
| Relevant evidence evaluation | ++ | + | **-** |

Mortality proportion: percentage of cases in which death was reported as an outcome in the overall cases report for a particular adverse event. IMEs and DMEs are developed and updated by EMA (European Medicines Agency, 2020). ++ : AEs are mainly from the FDA Prescribing Information, the Summary of Product Characteristics of esketamine posted by the MHRA, Phase 2/3 RCTs, or systematic reviews, with biological plausibility. + : AEs are mainly from other clinical trials, observational studies, or case reports/series with potential biological plausibility. - : AEs only emerging from disproportionality analyses.

AEs, Adverse Events; DMEs, Designated Medical Events; IMEs, Important Medical Events; MHRA, Medicine and Healthcare Products Regulatory Agency; RCTs, Randomized Controlled Trials; ROR_025_, the lower limit of 95% confidence interval of ROR.

**Supplementary Table S3**. Disproportionate distribution of positive signals of the pola+BR treatment protocol ( N < 10 ).

| PTs | N | ROR(95%CI) |
| --- | --- | --- |
| Ureteric compression | 9 | 796.47 (393.64-1611.51) |
| Magnetic resonance imaging spinal abnormal | 9 | 493.46 (248.42-980.20) |
| Eastern Cooperative Oncology Group performance status worsened | 9 | 52.18 (27.01-100.80) |
| Myelopathy | 9 | 48.14 (24.92-92.98) |
| Liver abscess | 9 | 34.78 (18.02-67.12) |
| Blood creatinine decreased | 9 | 30.14 (15.62-58.15) |
| Hepatitis B | 9 | 27.66 (14.34-53.35) |
| Monocyte count increased | 9 | 21.24 (11.02-40.95) |
| Red cell distribution width increased | 9 | 16.12 (8.36-31.07) |
| Neutrophil count increased | 9 | 10.50 (5.45-20.24) |
| Decreased activity | 9 | 7.70 (4.00-14.84) |
| Red blood cell count decreased | 9 | 3.21 (1.67-6.18) |
| Arrhythmia | 9 | 2.75 (1.43-5.30) |
| Liver disorder | 9 | 2.59 (1.34-4.99) |
| Hypercreatininaemia | 8 | 190.23 (93.75-386.00) |
| Procalcitonin increased | 8 | 63.61 (31.62-127.97) |
| Lymphadenopathy mediastinal | 8 | 56.32 (28.01-113.25) |
| Lymphocytosis | 8 | 50.09 (24.92-100.69) |
| Gastrointestinal obstruction | 8 | 37.51 (18.68-75.33) |
| Left ventricular failure | 8 | 33.08 (16.48-66.41) |
| Lung opacity | 8 | 21.74 (10.83-43.60) |
| Post transplant lymphoproliferative disorder | 8 | 16.74 (8.35-33.58) |
| Hypoalbuminaemia | 8 | 13.79 (6.88-27.64) |
| Hypogammaglobulinaemia | 8 | 12.75 (6.36-25.57) |
| Thrombotic microangiopathy | 8 | 10.29 (5.13-20.62) |
| Cardiomyopathy | 8 | 9.08 (4.53-18.21) |
| Hiatus hernia | 8 | 8.54 (4.26-17.12) |
| Acute respiratory failure | 8 | 5.20 (2.60-10.43) |
| Hepatic function abnormal | 8 | 2.72 (1.36-5.44) |
| Speech disorder | 8 | 2.05 (1.02-4.10) |
| Ilium fracture | 7 | 526.35 (241.26-1148.33) |
| Catheter site thrombosis | 7 | 213.73 (100.16-456.08) |
| Resorption bone increased | 7 | 177.21 (83.26-377.16) |
| Hilar lymphadenopathy | 7 | 108.51 (51.24-229.79) |
| Bacterial disease carrier | 7 | 93.79 (44.33-198.41) |
| Klebsiella sepsis | 7 | 88.38 (41.79-186.90) |
| Abdominal neoplasm | 7 | 88.16 (41.69-186.43) |
| Osteolysis | 7 | 49.60 (23.52-104.58) |
| Atrial tachycardia | 7 | 36.65 (17.40-77.22) |
| Lymphadenitis | 7 | 35.05 (16.64-73.83) |
| Cardiac dysfunction | 7 | 13.50 (6.42-28.40) |
| Bone marrow failure | 7 | 5.35 (2.54-11.24) |
| Scar | 7 | 5.18 (2.47-10.89) |
| Platelet count increased | 7 | 5.13 (2.44-10.79) |
| Pancreatitis acute | 7 | 4.95 (2.35-10.40) |
| Pneumonia escherichia | 6 | 299.09 (131.07-682.53) |
| Cystitis viral | 6 | 217.33 (95.84-492.81) |
| Alanine aminotransferase decreased | 6 | 103.45 (46.03-232.51) |
| Glomerular filtration rate increased | 6 | 62.41 (27.86-139.82) |
| Pneumonia pseudomonal | 6 | 36.93 (16.52-82.56) |
| Metastases to meninges | 6 | 32.13 (14.38-71.82) |
| Neck mass | 6 | 22.06 (9.88-49.27) |
| Hypotonia | 6 | 10.32 (4.62-23.02) |
| Hydronephrosis | 6 | 10.11 (4.53-22.56) |
| Pseudomonas infection | 6 | 8.61 (3.86-19.21) |
| Central nervous system lesion | 6 | 6.20 (2.78-13.83) |
| Splenomegaly | 6 | 5.86 (2.63-13.07) |
| Night sweats | 6 | 2.32 (1.04-5.18) |
| Drug-induced liver injury | 6 | 2.23 (1.00-4.98) |
| Human polyomavirus infection | 5 | 108.91 (44.84-264.50) |
| Pelvic venous thrombosis | 5 | 76.00 (31.38-184.06) |
| Pneumoperitoneum | 5 | 41.72 (17.28-100.73) |
| Post-acute COVID-19 syndrome | 5 | 34.27 (14.20-82.70) |
| Acute graft versus host disease in skin | 5 | 26.18 (10.86-63.12) |
| Escherichia urinary tract infection | 5 | 14.27 (5.92-34.37) |
| Metabolic disorder | 5 | 11.59 (4.81-27.90) |
| Pulmonary function test decreased | 5 | 11.20 (4.65-26.97) |
| Tumour lysis syndrome | 5 | 6.05 (2.51-14.56) |
| Blindness unilateral | 5 | 4.91 (2.04-11.81) |
| Eye infection | 5 | 4.47 (1.86-10.76) |
| Acute respiratory distress syndrome | 5 | 4.08 (1.69-9.81) |
| Ulcer | 5 | 3.71 (1.54-8.93) |
| Haematocrit decreased | 5 | 3.32 (1.38-8.00) |
| Transaminases increased | 5 | 2.86 (1.19-6.87) |
| Cholelithiasis | 5 | 2.43 (1.01-5.85) |
| Adenoviral encephalitis | 4 | 2514.24 (756.33-8358.05) |
| Kidney malrotation | 4 | 1117.44 (377.76-3305.48) |
| Abdominal incarcerated hernia | 4 | 591.58 (209.68-1669.05) |
| Retroperitoneal mass | 4 | 346.79 (125.75-956.36) |
| Tri-iodothyronine free decreased | 4 | 166.23 (61.32-450.63) |
| Morganella infection | 4 | 134.99 (49.95-364.85) |
| Retroperitoneal lymphadenopathy | 4 | 106.99 (39.69-288.38) |
| Aspartate aminotransferase decreased | 4 | 90.60 (33.66-243.83) |
| Superior vena cava syndrome | 4 | 79.50 (29.57-213.72) |
| Bone marrow infiltration | 4 | 76.48 (28.46-205.54) |
| Adenoma benign | 4 | 53.63 (20.00-143.83) |
| Enterococcal bacteraemia | 4 | 50.54 (18.85-135.48) |
| Hyperleukocytosis | 4 | 33.46 (12.50-89.57) |
| Osteosclerosis | 4 | 32.97 (12.32-88.24) |
| Hyperaemia | 4 | 29.32 (10.96-78.44) |
| Livedo reticularis | 4 | 29.32 (10.96-78.44) |
| Large intestinal ulcer | 4 | 18.30 (6.85-48.91) |
| B-cell lymphoma | 4 | 16.93 (6.34-45.23) |
| Neoplasm recurrence | 4 | 15.60 (5.84-41.68) |
| Cancer pain | 4 | 15.08 (5.64-40.28) |
| Creatinine renal clearance decreased | 4 | 12.59 (4.71-33.63) |
| Hypovolaemia | 4 | 11.90 (4.46-31.78) |
| Shock haemorrhagic | 4 | 6.66 (2.49-17.77) |
| Blood thyroid stimulating hormone increased | 4 | 6.43 (2.41-17.17) |
| Hepatomegaly | 4 | 6.22 (2.33-16.60) |
| Polyuria | 4 | 6.19 (2.32-16.52) |
| Escherichia infection | 4 | 6.02 (2.25-16.06) |
| Oesophagitis | 4 | 5.94 (2.23-15.86) |
| Cardiomegaly | 4 | 5.84 (2.19-15.60) |
| Ileus | 4 | 5.29 (1.98-14.11) |
| Clostridium difficile colitis | 4 | 5.03 (1.88-13.43) |
| Blood urea increased | 4 | 4.78 (1.79-12.76) |
| Bronchospasm | 4 | 4.48 (1.68-11.97) |
| Pneumocystis jirovecii pneumonia | 4 | 4.10 (1.53-10.93) |
| Coagulopathy | 4 | 4.08 (1.53-10.88) |
| Hypomagnesaemia | 4 | 3.54 (1.33-9.46) |
| Nodule | 4 | 3.20 (1.20-8.54) |
| Mass | 4 | 3.15 (1.18-8.42) |
| Hypocalcaemia | 4 | 2.76 (1.04-7.37) |
| Autologous haematopoietic stem cell transplant | 3 | 274.11 (85.66-877.09) |
| Blood lactate dehydrogenase decreased | 3 | 162.11 (51.29-512.35) |
| Cytomegalovirus hepatitis | 3 | 100.50 (32.02-315.49) |
| Sarcopenia | 3 | 74.63 (23.84-233.60) |
| Metastatic lymphoma | 3 | 73.54 (23.50-230.15) |
| Non-Hodgkin's lymphoma recurrent | 3 | 71.45 (22.84-223.55) |
| Clostridium colitis | 3 | 69.47 (22.21-217.33) |
| Humoral immune defect | 3 | 63.34 (20.26-198.01) |
| Choroidal neovascularisation | 3 | 55.02 (17.62-171.84) |
| Chylothorax | 3 | 53.08 (17.00-165.75) |
| Mechanical ileus | 3 | 49.43 (15.84-154.27) |
| Blood bilirubin decreased | 3 | 48.79 (15.63-152.27) |
| Perirectal abscess | 3 | 42.47 (13.62-132.44) |
| Blood immunoglobulin A decreased | 3 | 41.30 (13.24-128.80) |
| Pseudomonal bacteraemia | 3 | 38.56 (12.37-120.19) |
| C-reactive protein decreased | 3 | 28.12 (9.03-87.57) |
| Blood immunoglobulin M decreased | 3 | 25.12 (8.07-78.21) |
| Malignant pleural effusion | 3 | 23.85 (7.66-74.24) |
| Strongyloidiasis | 3 | 23.23 (7.46-72.29) |
| Pancreatitis chronic | 3 | 20.10 (6.46-62.53) |
| Neurological decompensation | 3 | 16.57 (5.33-51.51) |
| Systemic candida | 3 | 14.66 (4.72-45.59) |
| Escherichia sepsis | 3 | 14.55 (4.68-45.24) |
| Urinary tract obstruction | 3 | 13.92 (4.48-43.27) |
| Bacterial sepsis | 3 | 13.46 (4.33-41.84) |
| Coronavirus test positive | 3 | 13.01 (4.18-40.43) |
| Diabetic neuropathy | 3 | 11.31 (3.64-35.14) |
| Intestinal ischaemia | 3 | 8.25 (2.66-25.64) |
| Enterococcal infection | 3 | 7.73 (2.49-24.01) |
| Benign prostatic hyperplasia | 3 | 7.31 (2.35-22.71) |
| Renal function test abnormal | 3 | 6.96 (2.24-21.61) |
| Colitis ischaemic | 3 | 6.88 (2.21-21.37) |
| Klebsiella infection | 3 | 6.62 (2.13-20.56) |
| Atrioventricular block | 3 | 5.72 (1.84-17.75) |
| Metastasis | 3 | 5.33 (1.72-16.55) |
| Polyp | 3 | 4.49 (1.45-13.96) |
| General physical condition abnormal | 3 | 4.11 (1.32-12.77) |
| Generalised oedema | 3 | 3.92 (1.26-12.19) |
| Electrolyte imbalance | 3 | 3.34 (1.08-10.38) |
| Myelodysplastic syndrome | 3 | 3.32 (1.07-10.32) |

PTs , preferred terms; N, number of cases; ROR, Reporting Odds Ration; CI, Confidence Interval
